# Trying to Solve the Puzzle of the Interaction of Ascorbic Acid and Iron: Redox, Chelation and Therapeutic Implications

**DOI:** 10.3390/medicines7080045

**Published:** 2020-07-30

**Authors:** George J. Kontoghiorghes, Annita Kolnagou, Christina N. Kontoghiorghe, Loukia Mourouzidis, Viktor A. Timoshnikov, Nikolay E. Polyakov

**Affiliations:** 1 Postgraduate Research Institute of Science, Technology, Environment and Medicine, Limassol CY-3021, Cyprus; an.kolnagou@gmail.com (A.K.); xtina_jt@hotmail.com (C.N.K.); loukia.mourouzidi@gmail.com (L.M.); 2 Institute of Chemical Kinetics and Combustion, 630090 Novosibirsk, Russia; timoshnikov@kinetics.nsc.ru

**Keywords:** ascorbic acid, vitamin C, iron, chelation, nutrients, reactive oxygen species, antioxidants, therapeutics

## Abstract

Iron and ascorbic acid (vitamin C) are essential nutrients for the normal growth and development of humans, and their deficiency can result in serious diseases. Their interaction is of nutritional, physiological, pharmacological and toxicological interest, with major implications in health and disease. Millions of people are using pharmaceutical and nutraceutical preparations of these two nutrients, including ferrous ascorbate for the treatment of iron deficiency anaemia and ascorbate combination with deferoxamine for increasing iron excretion in iron overload. The main function and use of vitamin C is its antioxidant activity against reactive oxygen species, which are implicated in many diseases of free radical pathology, including biomolecular-, cellular- and tissue damage-related diseases, as well as cancer and ageing. Ascorbic acid and its metabolites, including the ascorbate anion and oxalate, have metal binding capacity and bind iron, copper and other metals. The biological roles of ascorbate as a vitamin are affected by metal complexation, in particular following binding with iron and copper. Ascorbate forms a complex with Fe^3+^ followed by reduction to Fe^2+^, which may potentiate free radical production. The biological and clinical activities of iron, ascorbate and the ascorbate–iron complex can also be affected by many nutrients and pharmaceutical preparations. Optimal therapeutic strategies of improved efficacy and lower toxicity could be designed for the use of ascorbate, iron and the iron–ascorbate complex in different clinical conditions based on their absorption, distribution, metabolism, excretion, toxicity (ADMET), pharmacokinetic, redox and other properties. Similar strategies could also be designed in relation to their interactions with food components and pharmaceuticals, as well as in relation to other aspects concerning personalized medicine.

## 1. Introduction

Iron and ascorbic acid or vitamin C, are two of the most essential nutrients that are required for normal growth and development in humans. The daily acquisition of sufficient quantities of these nutrients is important for normal physiological function and activity, and their absence or reduced body intake and tissue distribution could result in serious diseases. There are common pathways and interactions between iron and ascorbic acid metabolism, some of which could have an impact on normal living and in disease states. Similarly, since both nutrients are likely to be in the same daily meals, their chemical interactions are also of nutritional significance and interest.

Iron is essential for all living organisms, including microbes and cancer cells. There are many iron-containing proteins and metabolic pathways, which play an important role in cellular and physiological functions. There is also operational effective homeostatic control of the metabolic pathways associated with iron absorption, utilization, recycling and possibly excretion [1,2,3,4]. The continuous production and timely turnover of iron-containing proteins ensures normal biological and physiological activity. In this context, any abnormalities in the production of iron-containing proteins, the iron metabolic pathways and associated processes, could lead to different diseases [3,4,5,6].

Iron metabolic imbalance is generally associated with a number of serious conditions, such as iron deficiency anaemia, which affects about a third to a quarter of the world’s population [7]. In contrast, many hundreds of thousands of patients with chronic haematological and malignant diseases, including thalassaemia, sickle cell anaemia, haematopoietic stem cell transplantation, aplastic anaemia and cancer, are affected by iron overload due to multiple red blood cell transfusions for the treatment of the refractory anaemia [8,9,10,11]. Similarly, iron overload caused by increased gastrointestinal iron absorption in the inherited disease idiopathic haemochromatosis affects thousands of people, especially populations of Caucasian origin [12,13].

The regulation of iron absorption, transport, storage and utilization in humans is primarily controlled by a number of specific regulatory proteins and transcription factors [1,2,3,4,5,14]. Similar to other essential metal ions, iron has, under normal conditions, a specific metabolic route, biomolecular sites of interaction, specific tissues of deposition and storage and specific pathways of transport in blood and in cells. In this context, the body fluids and organs of normal individuals contain iron levels within a certain range, which reflect the requirements for normal bodily functions and the balance of dietary intake and excretion [4,5,6]. In general, the distribution of iron in the body of a 70–75 kg man is mainly in the form of haem-iron (Fe^2+^) in haemoglobin (2.3–2.6 g) in red blood cells, and of myoglobin (0.32–0.40 g) in muscle. Non haem-iron such as stored iron (Fe^3+^) is found in ferritin (0.7 g) and haemosiderin (0.3 g), and also non-haem enzymes (0.1 g), totaling to 1.1–1.5 g in liver, spleen, muscle and bone marrow. Smaller amounts of iron are found in mitochondrial cytochromes (17 mg), catalase (5 mg) and transferrin (4 mg) [1,4].

Iron metabolic balance can be disturbed and affected by genetic, environmental, nutritional, iatrogenic and other factors, and may also be associated with other changes such as organ damage, inflammation, disease, trauma, sport activities, dietary habits, pregnancy, etc. [4,5,6,13,14].

A major area affecting health and disease, which involves iron and ascorbic acid, is free radical pathology. Increased production of free radicals (FR) and reactive oxygen species (ROS) has been implicated in almost all diseases, including those associated with tissue damage, as well as in cancer and ageing [15,16,17,18,19,20]. In almost all biological systems, the catalysis of FR production, including the effects leading to oxidative stress toxicity, is carried out by iron, copper and enzymes containing these metals. In general, many organic compounds can influence the rate of FR production, including reducing agents and or chelators, such as ascorbic acid [16,20].

Ascorbic acid is the most common vitamin, which is widely used as a nutraceutical supplement throughout the world. It is one of the most important compounds in the human diet derived from plants and is found in most fruits and vegetables. The essential need for ascorbic acid in humans seems to stem from the inability to biosynthesize this molecule in the body. This inability, which is also shared by other primates, is due to a mutation that causes a lack of production of l-gulono-1,4-lactone oxidase, an important enzyme in the metabolic route of ascorbic acid biosynthesis.

**Figure 1:**
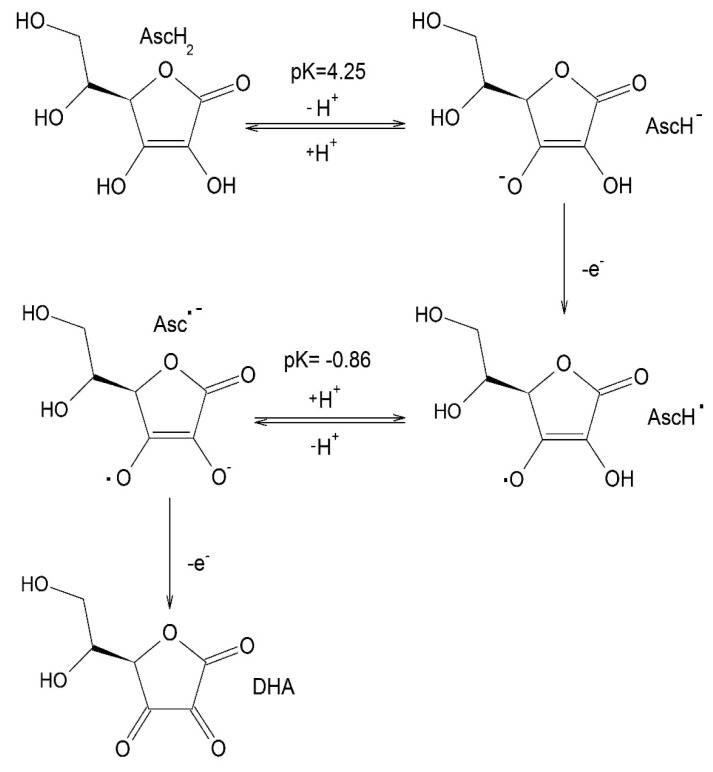
The structures of ascorbic acid (AscH_2_), ascorbate anion (AscH^−^) and its main oxidation products: ascorbyl radical in protonated and deprotonated forms (AscH^•^ or Asc^•^^−^) and dehydroascorbate (DHA) [31,32]. The metal binding site of ascorbic acid consists of the two –OH groups (one, 2-OH on the right hand site and the other 3-OH on the left hand site of the ring structure of AscH_2_) [32].

Ascorbic acid (AscH_2_) is mainly found in acidic conditions, e.g., the stomach environment, but the ascorbate anion (AscH^−^) predominates at physiological pH. Two other oxidation products of ascorbic acid, the ascorbyl radical (AscH^•^) and dehydroascorbate (DHA), are also found in the body in smaller quantities (Figure 1).

In addition to its nutritional value, ascorbic acid is also used in medical practice and in clinical trials for the treatment of many different conditions, including critically ill patients with hypovitaminosis C, in severe pneumonia, severe acute respiratory failure, multiple myeloma, metastatic colorectal carcinoma, metastatic melanoma, coronary artery disease, Type 2 diabetes, dementia, Alzheimer’s disease and COVID-19 [21,22,23,24,25,26,27,28,29,30].

The biological functions of ascorbic acid include antioxidant, chelating and coenzyme activities [33,34,35]. It is involved in tissue repair, the production of neurotransmitters, and is also important in the function of the immune system. The main property of ascorbic acid is its function as an antioxidant, and its antioxidant activity is usually associated with its ability to trap free radicals (R^•^), as shown in the reaction below:

AscH_2_ (or AscH^−^) + R^•^ → AscH^•^ (or Asc^•^^−^) + RH

As a powerful water soluble antioxidant, ascorbic acid acts as a scavenger of FR and provides effective protection to cell membranes, proteins and other biomolecules against oxidation by many ROS, including superoxide (O_2_^•−^), hydrogen peroxide (H_2_O_2_), hydroxyl radical (^•^OH), peroxyl radical (^•^OOH) and singlet oxygen (^1^O_2_) [16,17,18,36,37]. AscH^−^ donates a hydrogen atom (H^•^ or H^+^ + e^−^) to an oxidizing radical to produce the resonance-stabilized tricarbonyl ascorbate free radical (AscH^•^). However, AscH^•^ has a pKa of –0.86, and therefore is not protonated in physiological conditions and exists as Asc^•^**^−^**. The rate constants (*k*) for this reaction with the most common oxidants have been previously determined [38]. For example, the estimated *k* value for the tocopheroxyl radical (TO^•^) in a biological membrane at pH 7.4 is 2 × 10^5^ M^−1^s^−1^, and for hydroxyl radical (^•^OH), *k* = 1.1 × 10^10^ M^−1^s^−1^. AscH_2_ (or AscH^−^) reacts rapidly with TO^•^, ^•^OH and similar oxidants, making it an outstanding antioxidant.

In contrast to its antioxidant use and effects, ascorbic acid under certain conditions can also act as a pro-oxidant and a source of FR [39,40,41,42,43]. The nature of the pro-oxidant activity of ascorbic acid is related to its ability to reduce ferric iron via chelate complex formation, which is followed by the formation of ferrous iron and ascorbic radical. Since ferrous iron is involved in the process of ROS generation via the Fenton reaction, the reduction of ferric to ferrous iron will turn on the cyclic oxidation process. As a result, a significant increase in the oxidation rate of various organic compounds, including many biomolecules, through the Fenton reaction can be detected in the presence of ascorbic acid. In this context, the role of ascorbic acid in various diseases caused by iron and copper overloading conditions has been widely discussed [44,45,46]. Furthermore, there has been a heightened interest in the possibility of using ascorbic acid as a pro-oxidant for the treatment of cancer in combination with other anticancer agents, in high dose protocols of oral and intravenous administration [47,48,49,50,51,52,53,54,55,56,57,58].

Overall, there is continuous debate in the medical literature regarding the importance of iron and ascorbic acid and their effects and interactions, especially in relation to the treatment of many diseases. In this context, some of the molecular characteristics and interactions of iron and ascorbic acid are reviewed, with emphasis on the therapeutic implications in many categories of diseases affecting billions of patients. Major emphasis is also placed on the chemical mechanisms of the metabolic interactions between ascorbic acid and iron, in relation to the generation and inhibition of FR and ROS, as well as other metabolic aspects affecting their therapeutic applications and also their toxicity [15,16,17,18,19,20].

## 2. Historical Perspective of Ascorbic Acid

Ascorbic acid is found in various foods originating mainly from plants. The name “ascorbic acid” means “without scurvy”. It is used to prevent and treat scurvy, the disease caused by vitamin C deficiency. Scurvy was known to be a main killer of sailors during the long sea voyages, with apparent symptoms of skin bleeding, gum disease, hair changes, fatigue and anemia. Lind in 1753 in his Treatise on the Scurvy described how citrus fruits prevented the disease [59,60].

Ascorbic acid was discovered in 1912, and later in 1933 Haworth chemically identified the compound as l-hexuronic acid, proving this by chemical synthesis. Haworth and Szent-Györgyi proposed to name l-hexuronic acid as a-scorbic acid, and chemically l-ascorbic acid, in honor of its activity against scurvy. Szent-Györgyi and Haworth were awarded the 1937 Nobel Prizes in “Physiology and Medicine” and “Chemistry” respectively. The crystal structure of ascorbic acid was uncovered by Hvoslef only in the 1960s by neutron diffraction and X-ray spectroscopy [61,62].

In spite of the simplicity of this molecule (Figure 1), its biological role is still poorly understood due to its very complicated redox chemistry. The most intriguing property of ascorbic acid is its ability to interact with metal ions [20,63].

Ascorbic acid can be found in the World Health Organization’s List of Essential Medicines, as the safest and most effective medicine needed in a health system, and is available as an inexpensive synthetic or natural vitamin [64]. However, its multi-faceted role in human life and especially the molecular mechanisms of its biological activity are still under investigation, and the subject of many controversies and debates [65].

## 3. The Biological Redox and Toxic Effects of Iron

Iron is an essential element and important for human life due to its role in many cellular processes, including oxygen transport, energy transaction, DNA synthesis, and many metabolic processes involving iron-containing enzymes [1,2,3,4,5,6,16,39]. The catalytic activity of iron is related to its redox properties, namely the ability to alternate mainly between the two oxidation states Fe^3+^ and Fe^2+^, acting as an electron donor or acceptor [39,43].

Under normal conditions, there is a very low concentration of free, non-protein-bound iron in the human body. Iron (Fe^3+^) is transported in blood by plasma transferrin, which can bind and transport a maximum of two molecules of iron and deliver it to all cells of the body via transferrin receptors present at the cell surface [1,2,3,39]. All cells store iron using the intracellular storage protein ferritin, each molecule of which can store up to 4500 molecules of iron (Fe^3+^) in a polynuclear oxohydroxide complex formation. A low molecular weight iron pool is present intracellularly, which is composed of different natural chelators, including ascorbic acid, and is utilized for the exchange of iron between the different sites of uptake, removal and reuse [19]. Different mechanisms have also been suggested regarding the role of ascorbic acid in iron uptake and release from proteins of iron metabolism, including transferrin and ferritin [39,66,67].

Iron homeostasis is important for normal, healthy living [1,2,3]. The presence of excess iron is toxic to cells due to the involvement of high concentrations of free labile iron in various redox reactions, and the production of ROS via the Fenton type reactions, which can cause biomolecular, subcellular, cellular and tissue damage [68]. This is why the presence of excess iron is a negative prognostic factor, and can result in tissue damage in various diseases such as thalassaemia and other transfusional iron overload conditions, age-related neurodegenerative diseases and cancer [68,69,70,71,72,73,74].

**Figure 2:**
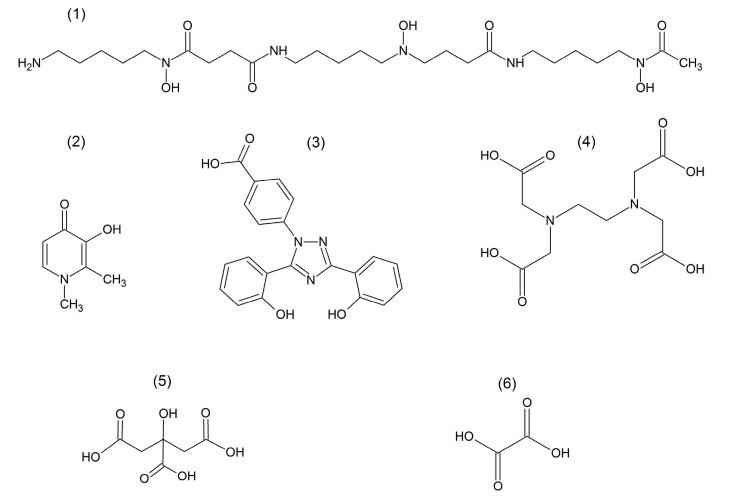
The chemical structures of the iron chelating drugs deferoxamine (1), deferiprone (2), deferasirox (3), ethylenediaminetetracetic acid (EDTA) (4), and the naturally occurring chelators citric acid (5) and oxalic acid (6).

Iron chelation therapy is widely used to prevent toxicity arising from the excess deposition of iron in tissues by removing the excess, toxic forms of iron, and preventing associated damage. Iron chelation therapy is carried out mainly by three widely used chelating drugs, deferoxamine, deferiprone and deferasirox, which have the ability to seek and bind excess iron and remove it from the body (Figure 2) [39,68,69,70,71,72,73,74,75,76,77].

The mode of action of the chelating drugs and other chelators and the mechanisms involved is the subject of many investigations. In particular, some very important questions have been raised in recent studies as to how the redox activity of iron changes in chelate complexes [20,75,78,79,80,81]. As an example, we have recently demonstrated the inhibition of iron- and copper-induced hydroxyl radical production by the water soluble chelator deferiprone (Figure 2) [81,82]. Deferiprone was discovered as an effective orally active iron-chelating drug developed for the treatment of iron overload toxicity in thalassaemia and other iron-related toxicity conditions [83,84,85]. It has also been demonstrated that deferiprone is an effective antioxidant that prevents oxidative stress and biomolecular, sub-cellular, cellular and tissue damage, caused mostly by iron- and copper-induced free radicals formation in vivo [85].

## 4. Iron Coordination and Redox Chemistry of Ascorbic Acid

The metal coordination chemistry of ascorbic acid has been thoroughly studied in connection with its high redox activity and participation in various biologically important redox processes [32,86,87,88,89,90,91]. In contrast to strong chelators, like the chelating drugs, which are used in iron overloading conditions for removing excess iron, ascorbic acid is generally considered as a weak chelating agent and cannot form strong iron complexes or be used effectively in iron removal. Being a weak dibasic acid (pKa1 = 4.1 and pKa2 = 11.79), ascorbic acid at physiological conditions exists as a monoanion (AscH^−^) with deprotonation of the 3-OH group (see Figure 1). This form is quite stable due to the delocalization of the negative charge among the oxygen atoms in the first and third positions. It has been previously suggested that the interaction of ascorbic acid with iron or other metal ions occurs by chelation via the O(3) and O(2) nuclei following hydrogen displacement from the 3-OH and the 2-OH groups (Figure 1) [32].

The chelate complexes of ascorbic acid with iron have been studied using different methods, including Mössbauer spectroscopy and time-resolved stopped-flow spectrophotometry techniques, where evidence for the presence of blue intermediates in the iron reduction pathway were observed during the reaction [86,92]. Furthermore, it has been demonstrated that the reaction proceeded in a millisecond time scale through an inner-sphere mechanism involving an Fe^3+^-chelate complex followed by the formation of an oxidation product, dehydroascorbic acid (DHA), in a number of steps (Figure 1) [86,92]. In these, as well as other investigations that followed, a fast reduction of Fe^3+^ via intramolecular electron transfer, with simultaneous production of ascorbyl radical, has been observed [80,81,82,83,84,85,86,87,88,89,90,91,92]. It has also been suggested that an iron chelate complex structure with stoichiometry 1:2 has been formed based on NMR data. A yellow Fe^2+^ chelate complex from methanol solution has also been isolated [93]. The data also provided evidence that the Fe^2+^ center in the chelate complex was in a distorted octahedral environment, realized in a polymeric structure, in which the ascorbate dianions used all their oxygen atoms as donor atoms. Further studies have also reported the formation of the mixed valence (Fe^2+^ and Fe^3+^) iron–ascorbate complexes [94].

**Figure 3:**
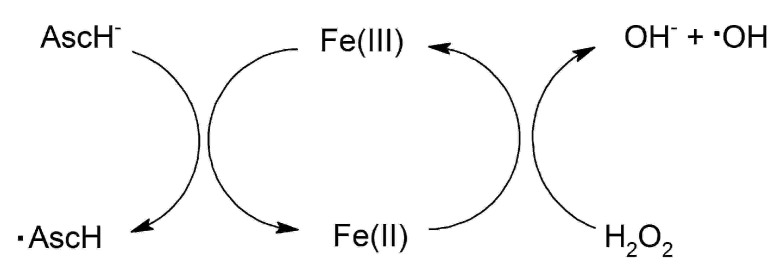
Ascorbate-driven cyclic Fenton reaction: schematic representation of the reduction of iron by ascorbate anion and the formation of the highly reactive hydroxyl radical (^•^OH) in the presence of hydrogen peroxide (H_2_O_2_).

Some of the main questions in relation to the formation of ferrous–ascorbate and ferric–ascorbate complexes are whether these complexes are redox active, and also whether there are any biological implications from such redox activity. In both cases, the initiation of a cascade of redox reactions with the formation of ROS is indicated, which is due to the ability of ascorbic acid to reduce Fe^3+^ via an intermediate chelate complex, producing a stable ascorbyl radical and the redox active Fe^2+^ which can lead to such activity (Figure 3).

The resultant resonance-stabilized ascorbate radical is stable, and it is eliminated by its interaction with reducing enzymes in order to regenerate the active form.

There are many examples describing the redox activity implications of a mixture of ascorbic acid with Fe^2+^ in the Fenton reaction [30,95,96,97,98]. For example, the oxidation of benzoic acid by the Fenton reaction occurs more effectively in the presence of ascorbic acid [96]. It appears that the starting reaction rate of the ferrous–ascorbate complex with hydrogen peroxide is the same as free Fe^2+^. However, after Fe^2+^ was oxidized to Fe^3+^, the reaction rate decreased by about one order. It was concluded that in the presence of ascorbic acid the starting reaction rate is kept high due, to the fast reduction of Fe^3+^ [96].

Similar redox-cycling behavior has been described earlier in previous studies, where the carotenoids beta-carotene and zeaxanthin, as well as anticancer quinine derivatives, were able to initiate the cycle of ROS generation in the presence of Fe^3+^ [97,98]. In general, it appears that redox cycling requires that iron in the form of an iron complex must first be reduced, and then oxidized by hydrogen peroxide. The thermodynamic rules for such redox-cycling have been formulated and proposed such that, for a metal-chelate to be an effective catalyst, it should have a reduction potential between E°(O_2_/O_2_^•–^) = −0.33 V and E°(H_2_O_2_, H^+^/HO^•^, H_2_O) = 0.46 V [99], or E°(H_2_O_2_, H^+^/HO^•^, H_2_O) = 0.39 V, as estimated and revised recently [100].

There is a lot of interest in redox cycling by metal chelates including iron chelate complexes in biological conditions and within cells. In this context, low molecular weight intracellular iron in a “transit pool” is considered to be bound and form complexes with low molecular weight naturally occurring endogenous chelators, such as amino acids, ATP, glutathione, citric acid, oxalic acid and dietary chelators such as ascorbic acid, polyphenols, etc. [29,39]. Considering that the iron concentration of the intracellular pool was estimated to be in the range of 1 µM, the formation of redox active complexes under certain conditions could be possible [101].

Redox active iron complexes could also be formed in vitro with low molecular weight natural chelators, such as citrate (0.1–0.15 mM, in plasma), in the presence of ascorbic acid and H_2_O_2_. Under these conditions, a mixed ascorbate iron citrate complex with an electrode potential of the Fe^3+^/Fe^2+^ citrate couple of about 0 V was reported, which could be redox active [102]. In contrast, the electrode potentials of the Fe^3+^/Fe^2+^ couple for strong complexes of iron chelators like deferiprone is near –0.6 V, and such complexes are not redox active [103]. The inhibition of hydroxyl radical generation in the Fenton reaction by deferiprone, deferoxamine and other chelators has been shown in much earlier studies, and was recently confirmed using an EPR spin trapping technique [20,81]. In this context, deferiprone and similar strong chelators could prevent oxidative stress toxicity during chelation therapy. The antioxidant effects of deferiprone in vitro, in vivo and under clinical conditions have been previously reviewed [85,104].

The transformation of ascorbic acid into several species (Figure 1) under different conditions, and as a result of redox and other interactions, may have different biochemical, toxicological and other implications, all of which are concentration-dependent. In this context, it has been suggested that under normal physiological conditions the plasma concentration of the ascorbate anion was estimated to be about 0.05 mM, which was much higher than the concentration of the ascorbyl radical [105]. Furthermore, it was also previously demonstrated that the electrode potential under physiological conditions for the ascorbyl radical/ascorbate anion couple is about +0.1 V, which is less than the standard potential of +0.28 V [106]. These results suggest that the ascorbate anion is far more likely to reduce “low molecular weight” chelate iron complexes than superoxide.

Some of the above mechanisms are thought to take place in plants, where iron is delivered to embryos as ferric complexes with citrate or malate [87]. In this context, it was suggested that embryos efflux high amounts of ascorbic acid that chemically reduce and liberate Fe^3+^ from Fe^3+^ chelate complexes. Overall, it was concluded that ascorbate ions play a key role in the chemical reduction and transport of Fe^2+^ within plant embryos. Similar iron transport mechanisms could be envisaged under certain conditions, intracellularly involving the low molecular weight iron pool and also present in plasma when transferrin is saturated with iron.

## 5. Biological Implications of the Iron Complexes of Ascorbic Acid

The alpha oxo-hydroxy and di-hydroxy ligands in the chemical structure of ascorbic acid are suitable for iron and other metal ion binding, as shown in a number of earlier studies on the formation of chelate complexes with Fe^2+^ and Fe^3+^ (Figure 1) [39,63,86,107,108].

There are many biological implications of the interactions of the ascorbate–iron complexes with cellular and sub-cellular components. In particular, the effect on iron transport through ascorbate–iron complexes in cells appears to be of physiological and toxicological importance. In this context, it was suggested that there are several routes of iron accumulation in cells, including the ascorbate-dependent ferrous iron uptake via the divalent metal transporter (DMT1), plus an independent route for ferric iron uptake [39,99]. For example, it was previously demonstrated that the reduction of ferric iron by ascorbic acid provides bioavailable ferrous iron to DMT1, and also directly enhances the uptake of ferric iron into Caco-2 cells through the formation of an Fe^3+^–ascorbate complex [109]. The Caco-2 cell line consists of human epithelial colorectal adenocarcinoma cells, which are generally used as an in vitro model of the human small intestinal mucosa to predict the absorption of orally administered drugs [109]. Two routes of iron accumulation have also been shown in studies with astrocytes, one involving the ascorbate-dependent ferrous iron uptake via DMT1, and the other an independent route for ferric iron uptake [110]. Since DMT1 is not involved in the uptake of ferric iron complexes, it is suspected that a different transporter system is in operation, similar to the mode of action observed by lipophilic chelators, which can facilitate the transport of ferric iron across cell membranes as previously shown in in vitro cell studies [111,112].

The interaction of ascorbic acid with other natural or synthetic chelators for iron and other metal complex formation is also of pharmacological and metabolic importance. It appears that under certain conditions, ascorbic acid can form mixed chelate complexes with other chelators. In previous studies, several examples of experimentally detected mixed ligand iron complexes of ascorbic acid, e.g., with phenol-amide and deferiprone, were reported [87,88,89,113]. Most of these mixed ligand iron complexes were shown to be redox active, but not in the case of deferiprone, which inhibits the pro-oxidant effects of ascorbate/iron [87,88,89,113,114]. Most of the redox active complexes appear to undergo fast interaction with the ascorbate anion to form ternary complexes, in which the ascorbate anion is bound to the Fe^3+^ center. Furthermore, these mixed complexes undergo intra-molecular electron transfer producing Fe^2+^ and DHA.

Studies of the structures and relative stability of ferric complexes with various chelators, including EDTA and ascorbic acid, have demonstrated that a neutral octahedral complex containing one iron atom and three ascorbate anions was found to be the most stable, but was still 92.7 kcal/mol less stable than the EDTA iron complex [90]. Nevertheless, in a clinical study the acute pro-oxidant effects of ascorbate during EDTA chelation therapy were detected when patients were administered an EDTA cocktail solution with 5 g of ascorbate [91]. Similar pro-oxidant effects of the ascorbate/EDTA combination were identified in earlier in vitro studies, where exacerbation of the breakdown of deoxyribose was observed in the presence of iron and hydrogen peroxide [20]. However, in other clinical studies, no such toxicity was observed when using a combination of EDTA and 7 g of ascorbate [115,116]. It should be noted that millions of patients are using daily combinations of EDTA and ascorbate in alternative medicine clinics worldwide, despite controversies over potential toxicity [117,118,119]. Overall, it appears from in vitro and clinical studies that under certain conditions EDTA may enhance the pro-oxidant and toxicity effects of ascorbic acid [9,20,118].

In contrast, in some cases the pro-oxidant activity of ascorbic acid can be considered a positive factor in cancer therapy, especially if used in combination with other anticancer agents such as the redox active quinone chelators [57,120,121]. The biomedical applications of iron–ascorbate complexes, including their potential as anti-tumor agents has been highlighted in a number of previous studies [49,50,51,52,53,54,55,56,57,58,68]. In particular, some investigators demonstrated biological plausibility, and are poised to explore the potential value of ascorbic acid in cancer treatment [54]. However, although not proven by the results of those studies, in general there is a clinically relevant positive effect of ascorbate supplementation in cancer patients on overall survival, clinical status, quality of life and performance status.

## 6. Toxicity Implications of the Interaction of Iron and Ascorbate in Physiological and Iron Loaded Conditions

The interactions of ascorbic acid, including iron binding, complex formation as well as the reduction of Fe^3+^ to Fe^2+^ in the absence and presence of other biomolecules, are multi-faceted processes, which are of great physiological and clinical importance. In particular, the interactions of iron with ascorbic acid in vivo, including the formation of metal complexes, could be affected by many other factors including the solubility and speciation of iron in physiological conditions.

Under normal physiological conditions, Fe^2+^ readily becomes oxidized to Fe^3+^ by atmospheric oxygen, and Fe^3+^ is hydrolyzed forming insoluble polymeric ferric oxyhydroxide complexes. Free, soluble aqueous Fe^3+^ is almost never found in detectable levels under physiological pH conditions (10^−18^ mol/L) in aqueous solutions, because of the high stability constant of the ferric oxyhydroxide complexes (log K = 38) [122,123,124,125,126]. Furthermore, transferrin can act as a ferroxidase by converting Fe^2+^ to Fe^3+^ and readily mobilizing any free, mononuclear Fe^3+^ and Fe^2+^ aqua, citrate, ascorbate and similar complexes in blood plasma [123,124,126,127,128,129,130,131,132,133]. Similar properties have been observed in the presence of the iron chelating drugs deferiprone and deferoxamine, and also in the case of lactoferrin, the sister protein of transferrin found in neutrophils and secretions such as milk, tears, etc. [20,81,113,124,133,134,135]. Subsequently to iron uptake, transferrin transfers Fe^3+^ to cells to be stored in ferritin or utilized in the production of haemoglobin and other iron-containing proteins, such as the iron–sulfur proteins [1,2,3,39,66,67,126,136,137,138,139,140].

There is a lot of debate and controversy regarding the presence, nature and toxicity of the “low molecular weight”, “labile” intracellular iron pool, and similarly of the non-transferrin-bound iron (NTBI) found in blood plasma mainly in cases of iron overload [141,142,143,144]. Under these conditions, it is suspected that NTBI is present in oligonuclear iron formation, and is also bound to albumin and to low molecular weight natural chelators such as citrate, phosphate and cysteine [142,143,144]. Furthermore, ascorbate, polyphenols and other dietary natural phytochelators, as well as many drugs with chelating properties, such as hydroxyurea, tetracyclines, doxorubicin, etc., are also expected to affect and have interactions with both the low molecular weight intracellular pool and also NTBI [145,146,147].

Regarding interactions under normal physiological conditions, both ascorbate and citrate have been suggested as major cofactors in iron metabolic pathways and as regulatory molecules of iron homeostasis [39,85,104,148,149]. In particular, ascorbate is thought to stimulate ferritin synthesis, inhibit lysosomal ferritin degradation, decrease cellular iron efflux and stimulate transferrin iron uptake synergistically with citrate in cells, via an intracellular reductive mechanism in the endosome (pH 5.6) [148,149]. Similarly, the biological activities of ascorbate and citrate also appear to play key roles in the development and progress of diseases related to iron metabolism [39,85,104,148,149].

There are many other biological implications of the interaction of ascorbic acid and iron, such as the prospect of increased production of ROS and toxicity in iron loading conditions, as well as the molecular modification and reduction or complete inactivation of other biological functions of ascorbic acid in its capacity as a major vitamin.

Non-transferrin-bound iron is well documented in iron overloaded diseases like thalassaemia, where transferrin is fully saturated with iron [70,142,143,144]. Variable amounts of NTBI are present in the plasma of iron-loaded thalassaemia patients, which have been previously estimated to be in the range between 0 and 25 μM [142,143,144,150,151]. The presence of labile, non-protein-bound iron is also considered as a source of continuous toxicity, which has been implicated in the pathogenesis of many other diseases, including atherosclerosis, neurodegenerative and kidney diseases, as well as cancer and ageing [68,70,144]. It appears that in general, the presence of ascorbic acid can exacerbate the toxicity of the intracellular labile “low molecular weight” iron, and of NTBI in the blood plasma of iron overloaded patients, by promoting the reduction of ferric iron to ferrous iron, which is capable of participating in the Fenton type redox reactions and which can lead to a vicious circle of molecular, sub-cellular, cellular and tissue damage [68]. Under these conditions, the extent of the damage caused by the iron/ascorbate combination depends on the rate of ROS production, but also on the rate and capacity of the innate antioxidant defenses and repair mechanisms [68].

However, despite the many reports of the toxicity of NTBI in experimental settings, its clinical significance in thalassaemia and other iron loaded conditions is questioned and is not utilized in clinical practice. The major concerns in relation to iron overloaded conditions associated with tissue damage, as well as the overall morbidity and mortality of iron loaded patients in clinical practice, are mostly associated with the evaluation of the level of excess iron deposition in major organs [10,11,12,152,153]. In this context, excess deposited iron, mainly in the form of haemosiderin, can be found in organs of iron loaded patients, including the heart, liver, spleen and pancreas, and monitored using magnetic resonance imaging (MRI) T2* relaxation times [154,155,156,157,158]. In most cases, organ damage in iron loaded conditions is directly related to the level of excess iron deposition, such as in the heart and liver of thalassaemia patients [158]. Similarly, excess iron or ‘focal’ iron deposits identified in the brain by MRI T2* have been implicated in neurodegenerative diseases, including Friedreich’s Ataxia, pantothenate kinase-associated neurodegeneration, Parkinson’s and Alzheimer’s diseases [126,159,160,161].

Different iron load toxicity ranges have been identified for specific organs using MRI T2* relaxation times [154,155,156,157,158]. For example, patients with cardiac MRI T2* relaxation times of lower than 8 ms are in the heavy haemosiderosis range and in danger of cardiac failure [158]. The cardiac MRI T2* relaxation times for patients with moderate cardiac iron overload is 8–12 ms, for mild iron overload 12–20 ms, and for normal individuals above 20 ms. Similarly, liver iron overload MRI T2* relaxation times of lower than 1.4 ms are considered to be in the severe hepatic haemosiderosis range, for moderate 1.4–2.7 ms, for mild 2.7–6.3 ms and for normal range above 6.3 ms [158,159,160,161].

Overall, it appears that in iron loaded diseases and also other diseases with focal iron deposits, the excess deposited iron in organs causes substantial damage and reduction in organ function, which may lead to irreversible damage [72]. In these cases, and also other diseases, the intracellular labile “low molecular weight” iron levels increase and can cause progressive ROS-related damage. Furthermore, the damage caused by iron can be potentially exacerbated by the presence of molecules such as ascorbate, or inhibited by other molecules such as deferiprone and other chelating drugs [20,81].

## 7. Nutritional and Vitamin C Functional Implications of the Interactions with Iron

The multi-role of vitamin C as an antioxidant at the molecular, sub-cellular, cellular and tissue level has been widely acknowledged in many studies. It appears that among many other antioxidant functions, vitamin C contributes to the maintenance of cellular redox homeostasis, protects the cell membrane from ROS and also protects and regenerates glutathione, vitamins A and E during oxidative stress. It is also a cofactor for a number of biosynthetic and gene regulatory enzymes, and participates in many metabolic pathways such as collagen formation, the metabolism of nor-epinephrine, the transformation of tryptophan to serotonin, the synthesis of carnitine, etc. Furthermore, vitamin C is involved in intracellular respiration, the reduction of cholesterol in blood, and the development of bone, teeth and cartilage structures, and also plays a major role in maintaining healthy immunity [162,163,164,165,166].

The interactions of iron with ascorbic acid, which are likely to affect many of the metabolic pathways and functions attributed to ascorbate’s role as a major vitamin, have not yet been fully elucidated. In particular, the structural changes of ascorbate due to oxidation by iron and the formation of the ascorbate radical and DHA may have general implications for its bioavailability and the reduction of many of its biological functions and roles. Many biochemical and clinical factors influence the impact of iron in each case, including the iron load levels, the nutritional status and the requirement levels for vitamin C, which differ in each individual.

The daily requirements of vitamin C, for example, vary among individuals and depend on age, gender, weight, physical activity, habits like smoking, and also general health status. It is estimated that the daily requirement for women is about 75 mg, for pregnant women 85 mg, for breastfeeding women 120 mg, for men 90 mg, and for teenage boys and girls 75 mg and 65 mg, respectively. An additional 35 mg per day is needed for smokers, and also different quantities for patients with different diseases, including patients with iron overload [164,166,167,168,169]. The major food sources of vitamin C are fruits and vegetables, and the highest amounts can be found in guavas, red peppers, kiwi, orange, green peppers, grapefruit, strawberries, melon, papaya, broccoli, peas, sweet potato, tomato, cauliflower and pineapple. The daily requirements for adult men and women can easily be covered with a few guavas, a large kiwi or an orange [165,168,169].

Vitamin C is readily absorbed from ingested food at large quantities of about 90%, of which about 50% is excreted. Its concentration in blood and bodily fluids does not increase above certain levels, despite increased intake [169]. In addition to scurvy, vitamin C deficiency can be observed in smokers, alcoholics, persons exposed to pollutants and radiation, and in many patient groups such as iron loaded patients, trauma, infectious diseases and cancer patients, where associated metabolic, antioxidant and physiological functions, including immunity, are affected. Higher consumption of vitamin C is recommended in these categories of patients for the alleviation of associated symptoms [162,164,165,170]. No major toxic side effects have been identified with high consumption of vitamin C, and the most common symptoms of excess consumption of about 2 g per day has been associated with gastrointestinal distress and diarrhea [165,168]. It should be noted that the safety findings of high doses of vitamin C apply only to different categories of patients with normal iron stores, and not to iron loaded patients, wherein toxic side effects may be observed due to an increase in oxidative stress from the interaction of excess iron with ascorbate [15,16,17].

There are many pathways and mechanisms of iron metabolism, in which ascorbic acid plays an important role in the maintenance of iron homeostasis, as well as in different interactions involving iron-containing proteins in health and disease [1,2,3,4,5,6,20,39,66,67,138,139]. It is estimated that total body iron in normal adults is about 4–5 g, and most of the iron is conserved and recycled, including the iron present in haemoglobin, which amounts to more than 60% of the total content. Only a few milligrams of iron are excreted or lost, and these are replaced from dietary iron sources [4,142,171,172,173]. The daily requirements for iron are different in each individual and depend on several parameters, including gender, age and stage of health [171,172,173,174,175]. For example, in adult men and post-menopause women, the daily requirement is 8 mg, for adult women 18 mg, for pregnant women 27 mg, for breastfeeding women 9–18 mg, and for teenage boys and girls 11 and 15 mg, respectively [162,171,174].

The rate of iron absorption in each individual is affected by many factors and their combination, including the quantity and quality (haem or non-haem) of dietary iron, the erythropoietic activity of the bone marrow, the presence of other dietary components such as natural chelators or drugs, reducing agents such as ascorbate, etc. [1,2,3,4,5,6,171,172,173,174]. Normally the absorption of iron in a western diet is about 2 mg/day, and equivalent losses allow the maintenance of body iron balance. Lower levels of absorbable iron are observed in vegetarian diets, which are mostly low in haem and in general can result in iron deficiency anemia, a condition widespread in vegetarian populations and malnourished patients [4].

## 8. Pharmacological Characteristics of Ascorbic Acid and Implications for Iron Metabolism

There are many common metabolic pathways in which iron and ascorbate are involved. Their interactions, including redox effects, could influence metabolic processes and may also have implications for health, as well as the causes or treatments of a variety of diseases. In each case the influence of either iron or ascorbic acid will depend on the mode of action, the specific properties and the characteristics of both of these nutrients, and their interactions with other molecules. Several parameters can influence the iron/ascorbate interactions, including their absorption, distribution, metabolism, excretion and toxicity (ADMET) characteristics, pharmacokinetics, and also other effects such as pH and concentration at the molecular, cellular and tissue level.

For example, both ascorbic acid and iron are required for a variety of biosynthetic pathways, particularly those involving hydroxylation reactions, including collagen biosynthesis, the hydroxylation of dopamine to nor-epinephrine, prolyl hydroxylation in hypoxic inducible factor (HIF), etc. [176]. Hydroxylases are iron-containing enzymes, and their activity also depends on iron regulation, which is a target for the treatment of related diseases, including those associated with collagen formation abnormalities in scurvy [39,177].

The implications of the interaction of the ascorbic acid metabolic by-products with iron are also concentration-dependent. Several of these ascorbic acid by-products appear to possess metal binding ligands, and to interact with iron under certain conditions [41,42,169]. The concentration of some of these chelating by-products appears to increase following excessive consumption or administration of ascorbic acid at high doses, and this may have an influence on iron and other metal metabolic pathways [169].

**Figure 4:**
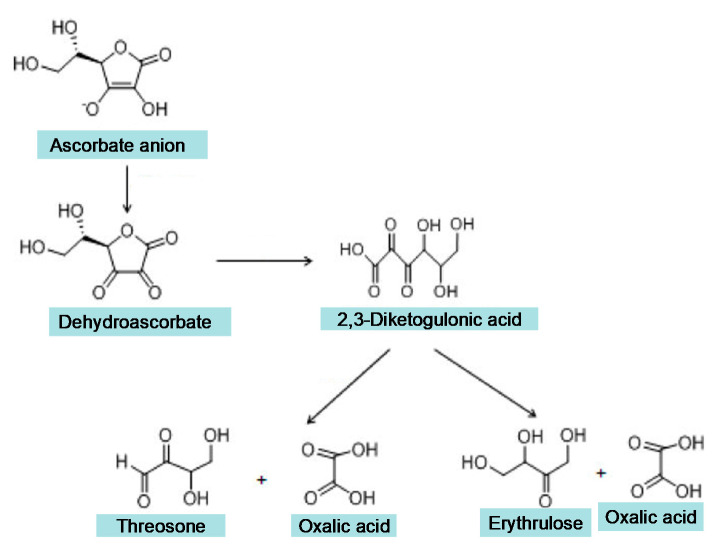
The non-enzymatic degradation of ascorbate to oxalate. Dehydroascorbate hydrolyses to 2,3-diketogulonic acid, which is then converted to erythrulose and oxalic acid. 2,3-Diketogulonic acid can also be converted to threosone and oxalate in the presence of hydrogen peroxide. All the degradation by-products, with the exception of dehydroascorbate, have ligands with iron and other metal binding potential. (Adapted from ref. [169]).

Different doses of ascorbic acid are generally used in clinical trials, and also by millions of people every day for antioxidant protection and other therapeutic effects. In addition to the molecular changes of ascorbic acid to ascorbate anion, ascorbic radical and DHA following redox reactions (Figure 1), oxalate is the major degradation, non-enzymatic breakdown product of ascorbic acid following oral and intravenous administration. The first step of ascorbic acid degradation to oxalate involves the formation of DHA, the second step the formation of 2,3-diketogulonic acid, and the final step the further breakdown of 2,3-diketogulonic acid to erythrulose, threosone and oxalic acid (Figure 4) [169]. It is estimated that about 50% of ascorbate is converted to oxalate and exclusively excreted in the urine. The amount of oxalate produced from ascorbic acid is about twice the amount produced normally from endogenous sources. It is estimated that the distribution of ascorbic acid and oxalate in a total of 15 L of plasma and extracellular compartments is 150 mg and 2.5 mg, in intracellular compartments of 25 L about 1.5 g and 50 mg, and in urine 20 mg and 30 mg, respectively [169]. Many reports suggest that particularly high oral or intravenous doses of ascorbic acid may cause oxalate nephropathy as a result of increased oxalate and oxalate–calcium complex formation [178]. It is also anticipated that under these conditions of high oxalate concentration, the metabolic pathways related to iron could also be affected, but no such observations have yet been studied or reported.

The wide range of oral and intravenous doses of ascorbic acid, and especially of high doses in clinical trials mainly in cancer patients, is indicative of its high tolerance and safety [47,48,51,52,53,179,180,181]. For example, doses of as much as 10 g/day intravenously for 10 days, and thereafter 10 g/day orally for several months, has been reported [182]. Similarly, in a pharmacokinetic study using doses of 5 to 60 g of ascorbic acid intravenously in 10 patients with metastatic prostate cancer for four weeks, a peak plasma concentration of 20.3 mM at the highest dose was observed, with elimination half-life of about 2 h at different doses [183]. The oral administration of different single doses of ascorbic acid in normal volunteers suggested that at 100 mg, none was excreted; the bioavailability was complete at 200 mg but declined at 500 mg or higher doses; and the absorbed amount was excreted, with oxalate and uric acid excretion increased as well, at 1000 mg doses, compared to lower doses [184]. At a dose of about 1200 mg, half was excreted in the urine. It was also estimated for doses of ascorbic acid of 200 mg and above that the plasma concentration was about 0.075 mM, and the concentration for cells of the immune system, i.e., neutrophils was 1.3 mM, monocytes 3.5 mM, and lymphocytes 3.2 mM. Decrease in serum ferritin was also observed during ascorbic acid treatments [184]. In another study ascorbic acid was found to be absorbed from the small intestine and to cross the blood brain barrier. There was variable distribution of ascorbic acid in organs and the highest concentration was reported to be present in the adrenal glands (550 mg/kg), the brain (140 mg/kg) and the liver (125 mg/kg) [185].

In relation to the metabolic and degradation products of ascorbic acid, with the exception of DHA, all other by-products, including 2,3-diketogulonic acid, erythrulose, threosone and oxalic acid, have iron chelation properties (Figure 4). Furthermore, despite the fact that DHA is not a chelator, it can be reverted back to ascorbic acid by reduced glutathione and reducing enzymes in some cells, like neutrophils and cells of the small intestine. In this context, the presence of ascorbic acid, metabolites and degradation products possessing chelating properties, not only in plasma but also intracellularly in blood cells and other cells of different tissues, can potentially affect the low molecular weight iron pool and many associated iron and redox metabolic processes. Physiological processes could also be affected under the same conditions, both in normal and disease states [4].

In general, it appears that ascorbate, metabolites and degradation by-products will be in competition with other natural chelators for iron or other metal complex formations, and will also participate in the formation of mixed iron and other metal complexes (Figure 1 and Figure 4). The affinity and concentration of these competing molecules are important parameters in iron complex formation and redox changes similar to other chelators [147]. For example, citrate (0.10–0.15 mM) in plasma, glutathione (5 mM) in liver cells and copper will affect redox and complex formation interactions between iron and ascorbic acid, as well as the ascorbic acid metabolites and by-products [147,186].

## 9. The Role of Ascorbic Acid in Iron Absorption and Iron Excretion

Ascorbic acid has been widely used as an antioxidant in many clinical trials in cancer and other diseases, such as sepsis and acute respiratory failure [21,22,23,24,25,26,27,28,29,30,47,48,51,52,53,179,180,181,187,188]. In most of these clinical studies, the implications of the molecular interactions of iron with ascorbic acid have not yet been fully characterized. However, most of the clinical evidence concerning the effects of the molecular interactions of iron and ascorbic acid can be obtained from the use of ascorbic acid in the treatment of diseases associated with iron metabolic imbalance, and in particular iron deficiency anaemia and iron overload.

There are several cases where the molecular interactions of ascorbic acid and iron have been utilized in medical practice. Of particular interest is ferrous ascorbate, which is the drug of choice for the treatment of iron deficiency anaemia, especially in developing countries like India. For example, doses of 3–6 mg/kg/day of ferrous ascorbate for 12 weeks in children have led to substantial increases in haemoglobin levels of about 4–5 g/dL, which was thus more efficient than other iron formulations including iron polymaltose and ferrous sulfate [189,190,191,192].

In contrast to iron deficiency, ascorbic acid is considered as a standard adjuvant therapy, widely used with iron chelation therapy in thalassaemia patients for increasing iron excretion, especially in combination with deferoxamine [193,194,195,196]. In general, ascorbate status is considered a major factor determining the route and level of iron excretion, and many related studies have demonstrated substantial increases in iron excretion following administration of the combination [193,194,195,196]. In one study in thalassaemia patients, ascorbic acid therapy was invariably associated with increased iron excretion after subcutaneous deferoxamine ranging from 24% to 245% [194]. Similar effects were observed in idiopathic haemochromatosis patients, where increasing doses of 0.2 to 2.0 g of ascorbic acid were shown to cause progressive increases in iron excretion. Furthermore, it was also confirmed that ascorbic acid is not effective in increasing iron excretion without the use of deferoxamine [197]. Current protocols for the optimization of iron excretion caused by deferoxamine in thalassaemia and other transfusional iron loaded conditions involve the oral administration of a total of 400 mg; 200 mg before and 200 mg during the subcutaneous infusion of deferoxamine.

Different mechanisms are involved in the ascorbic acid-induced changes of facilitated iron absorption in comparison to iron excretion. The promotion of non-haem iron gastrointestinal absorption by ascorbic acid seems to involve the partial reduction of Fe^3+^ to Fe^2+^, and the increased iron solubility of both Fe^2+^ and Fe^3+^ due to complex formations followed by enterocyte uptake. Only a partial intake of available iron is accomplished, because of factors including competition from other nutrients with chelating properties, and also other metals such as copper and zinc [147,198]. In this context, interference in iron absorption promoted by ascorbic acid can be influenced by many food components with metal binding ligands, including phytochelators such as polyphenols, phytic acid and tannins [39,199,200,201,202]. Furthermore, food components, similar to those above-mentioned, can enact redox activities in interactions with iron, which can also influence the absorption of iron promoted by ascorbic acid [97,98,110,203,204,205,206,207]. Under these conditions and influences, the optimization of iron absorption by ascorbic acid can be achieved by administration on an empty stomach, and preferably before meal intake.

The effect of ascorbic acid on the increase of Fe^3+^ absorption requires further investigation, considering the new developments in the area and in particular following the regulatory approval of ferric maltol (Feraccru) for the treatment of iron deficiency anaemia [6,39,198,207]. The original investigations and proposal for the use of ferric maltol in the treatment of iron deficiency anemia was reported almost 40 years ago [124].

## 10. Drug Interactions with Ascorbic Acid and Iron

There are many drug interactions between ascorbic acid and iron which go beyond the treatment of iron overload and iron deficiency anemia. In addition to interactions with food constituents, both ascorbic acid and iron can interact with different drugs, such as those with metal binding potential and redox activity, and also drug formulations containing metal ions [146]. In this context, redox, chelation and other forms of interactions between ascorbate and iron will apply, as previously discussed. These interactions will depend on the concentration of each component, the pharmacokinetic and other parameters of the drugs, and also other factors [4,6,83].

The ascorbic acid interactions with different oral, intramuscular and intravenous iron formulations are of pharmacological, toxicological and clinical importance [205,206]. There are many ferrous and ferric iron formulations available, such as oral ferrous sulfate, ferrous gluconate, ferric fumarate, ferric polymaltose, ferric maltol, ferric iron dextran, ferric iron sucrose, ferric gluconate, ferric saccharate, etc., used for treating many categories of iron-deficient patients [190,191,192,207]. While oral iron formulations may be beneficial for patients given the increase of iron absorption in the presence of ascorbic acid, there is no advantage to using vitamin C during intramuscular and intravenous administration of different iron formulations, given the increasing prospects of pro-oxidant and other toxicity [208,209]. Similarly, the biological and clinical implications of such an interaction, including vitamin C deficiency side effects, are expected to increase in iron loaded patients and other patient categories receiving iron formulations [194,195,196].

The interactions of iron, ascorbic acid and their combination are anticipated to affect the pharmacological and toxicological effects of other drugs shown to be associated with iron and ascorbic acid metabolism. In this context, the co-administration of iron, vitamin C and their combination is likely to affect the treatment of thousands of patients receiving drugs such as tetracycline, hydroxyurea, doxorubicin, aspirin, etc. [146,147]. Many such pharmacological and toxicological effects can be avoided or minimized by hetero-chronic administration of the individual drugs involved in the interactions. Furthermore, chelators such as deferiprone and deferoxamine can be used as antidotes for preventing the pro-oxidant effects of ascorbic acid [20,81,113].

## 11. Future Therapeutic Strategies and Health Implications of the Use of Vitamin C and Iron

New findings are continuously being presented, in addition to the thousands of investigations already reported in relation to ascorbate, iron and their interactions. The essentiality of both of these nutrients, and the implications of their interactions for health and disease states, has not yet been fully determined. Within this context, many patients of different ages and genders are still found to suffer from “scurvy”-related symptoms, including iron metabolism changes, both in developed and developing countries [210,211,212]. Similarly, many patients in developed and developing countries have been diagnosed with iron deficiency of varying etiologies, and are treated with different types of iron formulations, including iron ascorbate, in order to identify the optimal therapy [213,214].

There are many other areas wherein the role of ascorbate could be investigated in relation to iron metabolism, which may have possible implications in the treatment of related diseases. In this context, the interactions between the proteins and pathways of iron metabolism, involved with innate metabolic controls associated with the absorption, distribution and excretion of iron, warrant further investigation [1,2,3,4,5]. For example, in addition to the interactions with iron-containing proteins, such as transferrin and ferritin, the effects of ascorbate on several other regulatory proteins, including erythropoietin, caeruloplasmin, ferroportin and hepcidin, which do not bind or carry iron but are key regulators in the movement of iron in and out of cells, should also be thoroughly investigated [1,2,3,4,5,215,216,217,218,219,220]. In particular, the effects of ascorbate and the ascorbate–iron complex on the function of hepcidin, a peptide hormone produced in the liver playing a central role in mammalian iron homeostasis by mediating the effects of erythropoiesis, hypoxia, inflammation and iron load on the levels of circulating iron, would be of great interest. Furthermore, the involvement and impact of ascorbate and the ascorbate–iron complex with regards to the new emerging therapies and strategies for iron metabolic disorders that have been proposed based on hepcidin agonists, antagonists and modified structural products, may help to identify optimal therapies for different categories of patients [221,222,223].

The role of ascorbate in the high incidence of infections, and also in the increase of iron excretion in combination with deferoxamine in iron loaded patients, needs to be further explored and optimized, especially considering that ascorbic acid is oxidized to oxalate by excess iron in heavily iron loaded patients [9,10,193,194,195,196,224]. Although the current consensus suggests that iron excretion is not regulated, there are several observations that need to be further re-evaluated in order to identify related mechanisms. These include observational studies asserting that iron losses, including urinary iron excretion, are substantially reduced in iron-deficient individuals, and are substantially increased in iron loaded patients in comparison to normal individuals [150,195,225,226]. Similar questions have arisen as to how iron loaded transplanted thalassaemia patients could achieve steady reductions in body iron, and in some cases have even achieved normal iron body levels several years after transplantation, without the use of chelation therapy or venesection [4,11,227,228,229].

Vitamin C is widely utilized by the general public as a powerful antioxidant for the prevention of cancer and other diseases, and millions of people buy and take different formulations every day. The investigations into vitamin C as an anticancer agent continue in a variety of cancer patient categories, mainly by using high doses in combination with other anticancer drugs, showing encouraging results in some cases [24,25,26,47,48,49,50,51,52,53,179,180,181,182,183]. Another branch of investigations into anticancer activity includes the pro-oxidant iron and copper complexes of vitamin C— qualities previously shown with other chelator metal complexes [69,74,230,231,232,233]. In this context, the combination of vitamin C with chelators such as omadine, thiosemicarbazones and triapine may enhance their anticancer potential, and also reduce their overall toxicity [74,230,231,232,233].

Studies into the many factors that can influence the biological and clinical activity of ascorbic acid, iron and their complex are also under study. It appears that many of the interactions of iron and ascorbic acid, including complex formation and antioxidant and pro-oxidant effects, can be influenced by other metal ions such as copper and zinc, as well as natural organic compounds such as polyphenols, most of which have iron chelating and or redox active properties [39,199,201,232,233,234,235]. In such cases, the interactions may affect the efficacy and toxicity of monotherapy and combination therapies of vitamin C. Similar influences can be exerted by chelating drugs such as deferiprone, and also many other drugs with metal binding ligands but with weaker affinities for metals such as tetracycline and hydroxyurea. In each of these cases, the influences of other molecules may have positive or negative effects, e.g., the pro-oxidant activity of ascorbate with iron in the presence of hydrogen peroxide can be inhibited by deferiprone, and exacerbated by EDTA [20,91,236].

A major unexplored area of biological and clinical interest in relation to the physiological and therapeutic activity of ascorbic acid, which affects iron interactions, is the chemical structure/activity correlation of ascorbic acid metabolites and their biological and clinical implications. For example, the interactions of ascorbic acid, which is the predominant species with a neutral charge in the acidic conditions of the stomach, are different from those of ascorbate, an anion, negatively charged under the physiological conditions of neutral pH. Similarly, the other vitamin C metabolites, which are also formed under physiological conditions, such as DHA and degradation by-products such as oxalate, may have other actions including causing toxic side effects at high concentrations [178]. In this context, the concentration and reactivity of each metabolite will affect iron binding, iron metabolism, and also other related biological and clinical activities.

It is envisaged that in general, optimized therapeutic protocols could be designed to be used in each clinical condition based on the ADMET, pharmacokinetic and other characteristics of ascorbate, iron and iron–ascorbate complexes, as well as other aspects concerning personalized medicine. However, the prospects of carrying out such investigations are not in most cases feasible, since the development of such therapeutic strategies related to personalized medicine is based on commercial, as opposed to ethical or academic criteria [237,238].

## 12. Conclusions

Iron and ascorbic acid are two of the most common essential nutrients, which under normal conditions are in daily contact due to their occurrence in different foods and in most meals, as well as in their use as nutraceutical supplements and in many drug formulations used by millions of people. There are some common pathways in the metabolisms of vitamin C and iron, as well as different interactions between them at the molecular, sub-cellular and cellular level. Some of these interactions may have therapeutic and toxicity implications, which can affect millions of patients.

One of the main interactions of iron and ascorbic acid is the formation of weak Fe^2+^ and Fe^3+^ complexes, and the initiation of redox reactions. The redox changes resulting from the presence of iron can cause a reduction in the antioxidant capacity of ascorbic acid, and also promote its pro-oxidant effects. In this case, iron not only compromises the antioxidant activity of vitamin C, but under certain conditions and especially in the presence of hydrogen peroxide can initiate through the Fenton reaction the increased production of FR and ROS, which can lead to oxidative stress and a vicious circle of bio-molecular, sub-cellular, cellular and tissue damage. This toxicity bears the hallmark of free radical pathology, which has been identified in the tissue damage of almost all pathological conditions affecting major organs, and also cancer and ageing. Strong chelators, like deferiprone, can be used as antidotes, and in most cases inhibit this form of FR and ROS toxicity as well as preventing associated damage.

The major mode of action of vitamin C as an antioxidant is widely exploited in many circumstances, including enhancing immunity and preventing, as well as inhibiting, the proliferation of cancer cells. Despite the fact that the anticancer activity of vitamin C has not been proven, even when used at high doses, the toxic side effects still remain negligible.

Among the established clinical uses of vitamin C is its activity in relation to iron metabolic disorders. There are different therapeutic approaches and uses of ascorbate in relation to conditions involving iron, for example in the treatment of patients with iron deficiency anemia and iron overload. In this context, the use of the Fe^2+^ ascorbate complex appears to promote the gastrointestinal absorption of iron, and is widely used in many countries for the treatment of iron deficiency anemia. In contrast, oral ascorbate in combination with subcutaneous deferoxamine appears to cause an increase in the level of iron excretion in iron loaded patients, and is therefore widely used as an adjuvant in chelation therapy in thalassaemia and other iron loaded patients. The monitoring of ascorbate levels is recommended for different categories of patients, such as the iron and copper overload, cancer chemotherapy and radiotherapy, immunocompromised and infectious disease groups of patients, etc., wherein suboptimal levels of ascorbate may affect the outcome of different treatments. The monitoring of oxalate levels is also recommended for patients receiving high doses of ascorbate to avoid possible excess oxalate-related toxic side effects.

The wide use of ascorbate and iron as nutraceutical supplements by millions of people, without therapeutic protocols or any clear guidelines concerning the possible negative interactions between these two nutrients, may lead to sub-optimal clinical effects and also toxic side effects. In this context, special recommendations apply to different categories of users of these nutrients, such as the recommended co-administration for increasing iron absorption, the avoidance of multi vitamin and mineral formulations where other metals may interfere with iron absorption, and the hetero-chronic administration of iron and ascorbate for optimizing the antioxidant activity of ascorbate.

Further work is recommended in order to optimize the therapeutic potential and minimize the possible toxicity implications of the use of ascorbate, iron and their combination in different diseases.
